# Spasticity and Its Contribution to Hypertonia in Cerebral Palsy

**DOI:** 10.1155/2015/317047

**Published:** 2015-01-11

**Authors:** Lynn Bar-On, Guy Molenaers, Erwin Aertbeliën, Anja Van Campenhout, Hilde Feys, Bart Nuttin, Kaat Desloovere

**Affiliations:** ^1^KU Leuven Department of Rehabilitation Sciences, 3000 Leuven, Belgium; ^2^Clinical Motion Analysis Laboratory, University Hospital Leuven, 3212 Pellenberg, Belgium; ^3^KU Leuven Department of Development and Regeneration, 3000 Leuven, Belgium; ^4^Department of Orthopedics, University Hospital Leuven, 3212 Pellenberg, Belgium; ^5^KU Leuven Department of Mechanical Engineering, 3000 Leuven, Belgium; ^6^KU Leuven Department of Neurosciences, 3000 Leuven, Belgium; ^7^Department of Neurosurgery, University Hospital Leuven, 3000 Leuven, Belgium

## Abstract

Spasticity is considered an important neural contributor to muscle hypertonia in children with cerebral palsy (CP). It is most often treated with antispasticity medication, such as Botulinum Toxin-A. However, treatment response is highly variable. Part of this variability may be due to the inability of clinical tests to differentiate between the neural (e.g., spasticity) and nonneural (e.g., soft tissue properties) contributions to hypertonia, leading to the terms “spasticity” and “hypertonia” often being used interchangeably. Recent advancements in instrumented spasticity assessments offer objective measurement methods for distinction and quantification of hypertonia components. These methods can be applied in clinical settings and their results used to fine-tune and improve treatment. We reviewed current advancements and new insights with respect to quantifying spasticity and its contribution to muscle hypertonia in children with CP. First, we revisit what is known about spasticity in children with CP, including the various definitions and its pathophysiology. Second, we summarize the state of the art on instrumented spasticity assessment in CP and review the parameters developed to quantify the neural and nonneural components of hypertonia. Lastly, the impact these quantitative parameters have on clinical decision-making is considered and recommendations for future clinical and research investigations are discussed.

## 1. Introduction

Muscle tone regulation helps to maintain normal posture and to facilitate movement [1]. When a muscle stretches, the neuromuscular system may respond by automatically altering muscle tone. This modulation of the* stretch reflex* is important in the control of motion and balance maintenance [2]. Spasticity is manifested by increased stretch reflex which is intensified with movement velocity [3]. This results in excessive and inappropriate muscle activation which can contribute to muscle hypertonia. Spasticity is a known impairment following an upper motor neuron (UMN) lesion, such as cerebral palsy (CP). In CP, spasticity is often regarded to be the most common motor impairment [4]. However, there are many uncertainties regarding the contribution of spasticity to hypertonia and, in particular, its contribution to the gait abnormalities seen in CP.

**Figure 1:**
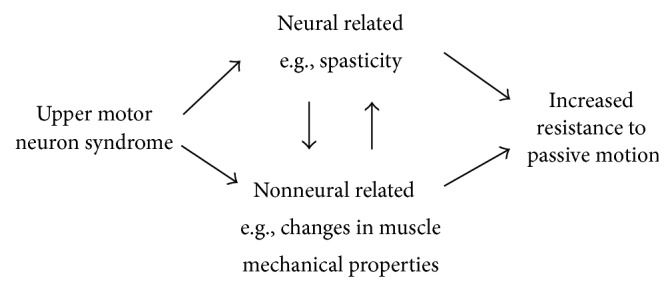
Neural and nonneural mechanisms contributing to increased resistance to passive motion in an upper motor neuron syndrome.

Much of this uncertainty is related to the miscommunication regarding the definition and assessment of spasticity. In clinical terms, hypertonia is assessed as the “resistance to passive stretch while the patient maintains a relaxed state of muscle activity” [5]. With spasticity-related hypertonia, lack of modulation of the stretch reflex causes premature and/or exaggerated muscle contraction that may resist the passive stretch. During clinical assessments, different stretch velocities can be incorporated and the increase in stretch reflex due to velocity is thereby subjectively evaluated. In reality, this clinical interpretation oversimplifies the fundamental physiological mechanisms of spasticity. Firstly, it is dependent on the reliance of the subjective interpretation of an examiner; secondly, the velocity of the stretch and level of relaxation of the muscle are uncontrolled; and thirdly, it does not allow differentiating between the contributions of neural and nonneural components to the overall resistance felt while stretching the muscle [6]. Nonneural mechanical muscle properties such as stiffness and viscosity are often altered in children with CP [7] and can also contribute to the feeling of increased resistance to passive motion (Figure 1).

Instrumented spasticity assessments are clearly more objective and valid than the clinical spasticity scales but have mostly been developed for adults and have received less attention in children with CP [8, 9]. Continued subjective evaluations of hypertonia in children with CP can lead to inaccurate management and ignorance of the necessity to distinguish between neural and nonneural components. For example, if spasticity contributes more to joint resistance than muscle stiffness, antispasticity medication is required, while, in case of predominance of stiffness over spasticity, options such as casting and orthotic management are more likely to be effective. Moreover, objective measurements allow for improved standardization between different assessors and clinical centers and increase the discrimination power between patients, providing better means to evaluate and direct treatment.

In this review article we firstly revisit what is known about spasticity in CP, including its definitions and its pathophysiology. Second, we summarize the state of the art on instrumented spasticity assessment in this population and review the parameters developed to quantify its contribution to muscle hypertonia. Third, we consider the impact of quantification of these parameters on clinical decision-making and discuss recommendations for future clinical and research investigations.

## 2. Cerebral Palsy and Spasticity

Three main subtypes of CP are based on the main motor disorder: spastic, dyskinetic, and ataxic [4]. All forms are characterized by abnormal posture or movement. In addition, spastic CP, known as a pyramidal motor disorder [5], is also characterized by hypertonia and/or pathological reflex activation [4]. In contrast, dyskinetic and ataxic forms of CP are thought to mostly arise from damage to the basal ganglia and cerebellum, respectively, and cause different movement abnormalities.

Spastic CP is the most commonly diagnosed disorder among children with CP [4]. Spasticity can affect the entire body, but it is generally worse in the lower limbs of children with bilateral involvement and in the upper limbs of children with unilateral involvement [10]. Spasticity of the trunk muscles can cause postural problems while spasticity of bulbar origin can result in difficulty in feeding and communication [11]. The most commonly affected lower limb muscles in children with CP are gastroc-soleus, hamstrings, rectus femoris, adductors, and psoas. In the upper limb, spasticity is most frequently found in the shoulder external rotators, elbow, wrist and finger flexors, and the elbow pronators [12]. Spasticity is thought to interfere with voluntary control and to increase energy consumption during movement [13]. Additionally, it hampers normal muscle lengthening during growth and is thus thought to contribute to the development of secondary muscle and soft tissue contractures and to skeletal deformation [14]. Muscle contractures and skeletal deformations can result in distorted internal and/or external lever arms resulting in abnormal joint moments during gait (lever-arm dysfunctions) [13].

## 3. Spasticity Definitions

There has been much debate regarding the definition of spasticity. This is mostly due to the term being used to refer to a general motor disorder rather than to a specific entity. The definition of spasticity by Lance (1980) as “a velocity dependent increase in stretch reflex” [3] gained popularity as it provided a specific pathophysiological rationale while acknowledging that spasticity was only one of the many features of an UMN syndrome. Other proprioceptive, cutaneous [15], and nociceptive [16] reflex circuits can also be affected by an UMN syndrome and can contribute to similar positive features (clonus, flexor spasms, clasp-knife phenomenon, and static tonic stretch reflex [11, 17]).

In 2003, the North American Task Force for Childhood Motor Disorders suggested that spasticity should be redefined as “a velocity dependent increase in hypertonia with a catch when a threshold is exceeded” [5]. Although hypertonia is a common clinical term, the inability of clinical scales to differentiate between the neural and nonneural components of increased resistance has led to the terms “spasticity” and “hypertonia” often being used interchangeably [18].

Outside a research setting, it is often impossible to isolate spasticity. Therefore, in clinical settings, different features are commonly combined and spasticity is referred to in a broader sense. In 2005, a European Thematic Network to Develop Standardized Measures of Spasticity (the SPASM consortium) suggested that the definition of spasticity should reflect a more clinical reality, and therefore it broadened its definition. They defined spasticity as “disordered sensory-motor control, resulting from an upper motor neuron lesion, presenting as intermittent or sustained involuntary activation of muscles” [19].

Both the previously mentioned narrow definitions and the latter wider approach have their disadvantages. When translating research findings to the clinic, the narrow definition results in a compromise on internal validity due to the inability to isolate spasticity. On the other hand, a broad definition hinders the development of targeted treatments. Understanding the underlying pathophysiology can help to create a distinction between spasticity and the other positive features. Rather than compromising and broadening the definition, efforts should be directed at effectively isolating and measuring the phenomenon in a clinical setting.

Another reason for the lack of agreement and on-going debate surrounding the definition of spasticity is the emerging evidence that spasticity is manifested differently in an active versus passive muscle [20]. With the exception of the definition by the SPASM consortium, spasticity has always been described by the levels of hyperreflexia or hypertonia when the muscle is at rest. Since testing muscle tone during active movement is technically very challenging, these definitions are, in part, a reflection of feasibility.

## 4. Pathophysiology of Spasticity

**Figure 2:**
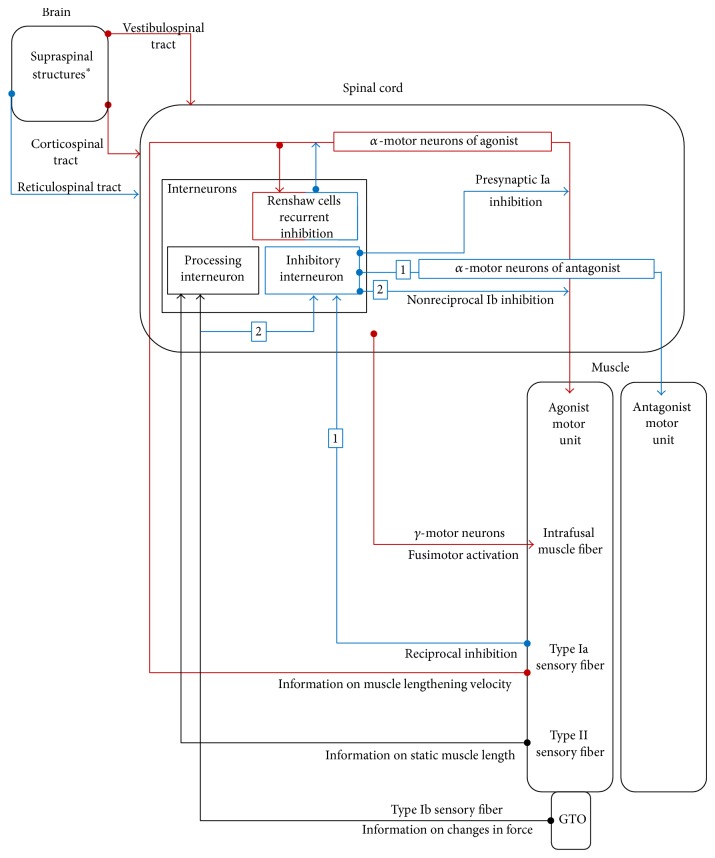
The major excitatory (red lines), inhibitory (blue lines), and processing (black lines) pathways involved in the reflex regulation contributing to normal trunk and limb muscle tone. Pathways numbered (1) and (2) travel first through interneurons before synapsing with alpha- (*α*-) motor neurons. GTO: Golgi tendon organ. ^*^Some extrapyramidal tracts (not shown in this figure) also contribute to the maintenance of normal muscle tone and not all pathways shown are necessarily involved in increased stretch reflex due to spasticity.

Spasticity is not caused by a single mechanism but rather by intricate changes along different interdependent pathways [2]. Given the complexity involved in regulating normal muscle tone, an UMN lesion can cause spasticity via many different pathways (Figure 2). Additionally, the mechanisms are dependent on etiology, location, and timing of the UMN lesion [21, 22].

The pathophysiology of spasticity has mostly been investigated through animal models and adult pathologies [2, 15]. In general, loss of typical control occurs due to deregulation of the motor pathways (mainly the corticospinal, reticulospinal, and the vestibulospinal tracts) running from the cerebral cortex and brain stem to the spinal cord [23]. Spasticity is not evident in lesions that affect only the corticospinal tract in the medullary pyramids or spinal cord [11]. Instead, damage to tracts that interact with the corticospinal tract is thought to contribute to spasticity. For example, damage along the reticulospinal tract decreases its inhibitory influence, resulting in increased muscle tone [15]. Loss of vestibulospinal tract excitation by the cortex is thought to cause decreased firing of the motor neurons, resulting in decreased extensor tone and thus a flexed posture. Other descending tracts thought to affect the regulation of stretch reflexes are the rubrospinal tract and the coerulospinal tract [11]. Further adaptations in the spinal networks as a result of the primary lesion are also thought to contribute to spasticity [15]. The main inhibitory spinal mechanisms thought to be involved in spasticity include reciprocal inhibition [23] and homosynaptic depression (also referred to as postactivation depression) [15]. While studies by Nielsen et al. (1995) demonstrated that decreased presynaptic inhibition played a role in spasticity in people with multiple sclerosis and spinal cord injury [21], in spasticity due to stroke, it seems not to be a systematic contributor [22, 24]. Recurrent Renshaw cell inhibition and Ib inhibition (Figure 2) are additionally thought to be decreased in muscles with spasticity [11]. The main excitatory mechanisms found to be related to spasticity in chronic spinal cord injury [25] and in persons after stroke [26] are plateau potentials [27] and enhanced cutaneous reflexes [15]. There is limited evidence of increased fusimotor drive [23]. Lastly, spasticity may also be aggravated by changes in the mechanical properties of muscles [28, 29], although little experimental evidence is present [27].

Unlike the pathology in adults whose motor system is developed at the time of injury, spasticity in children who suffer from an early brain abnormality is affected by reorganization of supraspinal input and impaired motor maturation. How the pathophysiology of spasticity is affected by maturation is an area of research which remains in its infancy of investigation.

## 5. Quantitative Measurement of Spasticity

In the last years, progress has been made to translate objective, instrumented spasticity measurement systems to clinical practice [6, 30–32]. These systems may still fall short of being able to distinguish between the different pathophysiological mechanisms of spasticity, but they have been shown to be more reliable and valid than the subjective clinical scales currently being used in most clinical settings [6, 33–38]. The most thorough set of reviews on spasticity assessments were carried out by the SPASM consortium in 2005 [39–41] and, more recently, also by our own group [8].

### 5.1. Passive Muscle Assessments

#### 5.1.1. The Neural Component

Approaches to assess spasticity when the muscle is at rest can be divided into clinical qualitative approaches and instrumented quantitative approaches. Quantitative approaches can further be divided into those methods that assess the neurophysiological response and those that assess the biomechanical response. Neurophysiological assessments help to quantify elevated reflex responses. A commonly assessed example is the Hoffman reflex (H-reflex), elicited by low threshold electrical stimulation of a mixed peripheral nerve. Alternatively, a tendon tap will elicit the tendon reflex (T-reflex), which follows a similar pathway to that of the H-reflex, but may also include the stretch reflex. Higher stimulation intensity of the mixed peripheral nerve results in the production of an M-wave and the eventual disappearance of the H-reflex. Lower H- and T-reflex latencies and higher reflex amplitudes are indicative of increased *α*-motor neuron excitability. The ratio of M-wave and reflex amplitudes (H_max⁡_/M_max⁡_ and T_max⁡_/M_max⁡_) has been used as a measure of spasticity. However, there is much overlap in the values of these ratios between healthy and spastic muscles, reducing their diagnostic ability [39]. Eliciting M_max⁡_ also requires a supramaximal stimulation, which is uncomfortable and therefore rarely used in children.

Alternatively, systems based on evoking a stretch reflex can evaluate both the neurophysiological and biomechanical behavior of muscles, joints, and limb segments, for example, by making use of electromyography (EMG) and simultaneous recording of angular velocity and torques during various, well-defined conditions (such as passive oscillations or ramp-and-hold stretches) when the muscle is at rest [39, 40]. In such methods, a distinction can be made between robotic designs, where the passive limb is manipulated by a motor-driven system, and manual designs, where an examiner applies a muscle stretch.

For eliciting the stretch reflex during passive movement, highly sophisticated, motor-driven devices are the most accurate in standardizing and controlling joint trajectory and movement velocity. Modeling the behavior of muscles to such systems provides insight into different components of increased resistance to passive motion [42, 43]. However, these methods are impractical for clinical use, especially in pediatric populations [41]. Alternatively, several manually controlled methods that integrate signals have also been developed [6, 30, 44–46]. These methods highly resemble the clinical assessment scales but additionally collect quantitative data using synchronized instruments. Since test performance will influence any collected signal, such tests require strict standardization. Additionally, thorough investigation of the psychometric properties of outcome parameters is essential prior to any clinical application. We recently reviewed the available manually controlled instrumented spasticity assessments and found that, to date, there is a paucity in methods that have been validated for use in children with CP [8].

Therefore, in a number of recent studies, we have explored the clinical relevance of several quantified parameters collected with a manually controlled instrumented spasticity assessment (ISA), developed for the lower limb muscles of children with CP [30, 34, 47–50]. ISA involves the simultaneous collections of EMG, velocity, and torque signals during ramp-and-hold passive muscle stretches at different velocities. The extracted parameters are compared between stretch velocities and include the amount of hyperactivation (average root mean square-EMG) [30, 48], the degree of hypersensitivity to activation (the spastic threshold) [49], the presence, location, and severity of a spastic catch [47], the type of muscle activation pattern (phasic or tonic) [49], joint torque at a particular angle, and work (the integral of torque versus position) [48]. A similar application for the elbow flexors has been validated for children with CP [51].

Although a measurement method based on passive movement can result in muscle activation by different cutaneous and proprioceptive reflex loops, the developed EMG parameters can be presumed to capture the electrophysiological responses evoked by velocity-dependent afferent input. As such, they reflect the definition of spasticity as offered by Lance [3]. The developed biomechanical parameters follow the same reasoning. They represent the velocity-dependent increase in the resistance to movement and are validated by a simultaneously occurring gain in the EMG signal. However, the exact relationship between evoked EMG and force production is not straightforward. Torque-related biomechanical parameters collected during passive stretch have proved to be less sensitive to the construct of spasticity than the simultaneously collected EMG-related parameters [30, 39, 44]. In the medial hamstring and gastroc-soleus, our studies showed less response to antispasticity medication, Botulinum Toxin-A (BTX), in the torque-, compared to the EMG-related parameters [34, 48, 50]. These findings suggest that the developed torque-related parameters do not adequately capture the contribution of spasticity to hypertonia. To do so, a more sophisticated decomposition of the torque signal is required.

Several torque decomposition models have been developed to better understand hypertonia [37, 38, 42, 52, 53], although only few have been applied in CP [43, 54]. The most straightforward of these models describe only the behavior of the nonneural components of hypertonia, such as passive stiffness [55] and viscosity [56]. These nonneural components have been studied in both healthy and hemiplegic subjects and are well described by polynomial or exponential mathematical models [42, 55]. More sophisticated mathematical algorithms have additionally modeled the neural contribution to the resistance measured during passive stretch [42, 43, 52]. de Gooijer-van de Groep et al. reported that reflex-related torque in the plantar flexors of children with CP was almost six times higher and tissue stiffness double that of controls [43]. Contradictory findings were found by Willerslev-Olsen et al. where the majority of assessed soleus muscles exhibited abnormal nonneural related stiffness, but in only a minority was reflex-related torque higher than that of controls [54]. These contradictions may be a reflection of different perturbation methods (six-degree movements [43] versus ramp-and-hold rotations over the entire range of motion [54]) and different torque decomposition models. Additionally, while de Gooijer-van de Groep et al. included all three plantar flexors, Willerslev-Olsen et al. analyzed only the soleus. Both articles reported a large variation in the ratio of neural and nonneural components in their sample, which emphasizes the need to individually define these components.

Most decomposition models have only ever been validated on data collected with motor-driven systems in which the displacement and/or the force applied can be well controlled. More recently, our group has applied a simpler method to extract the neural component based on measurements from the manually controlled ISA [50]. A simple model that describes stiffness and viscosity in healthy muscle was approximated from the algorithms introduced by de Vlugt et al. (2010) [42]. This model was fitted to the torque-position data collected at the ankle during low velocity full range of motion manipulations. The model was then fitted to stretches in which a stretch reflex was evoked (high velocity stretches). The amount of deviation between the modeled and measured torque during these latter stretches represented the pathological neural component. This “deviation-parameter” was found to be repeatable between assessments and to distinguish between healthy and spastic muscle. Additionally, unlike the previously described torque-related parameters containing both neural and nonneural components, the deviation-parameter was found to decrease after BTX [50].

This may be the first step towards breaking down the measured torque into a clinically relevant representation of the contribution of spasticity to hypertonia. Unfortunately, this method could not be used to accurately estimate the amount of stiffness and viscosity nor to separate the properties of muscles from the properties of tendons (see discussion in [50]). To achieve this, a combination of muscle imaging and modeling work is required and is scope for further study.

When quantifying the neural component, it is also important to consider that different muscles possess different activation patterns and different biomechanical properties. Consequently, the effect of spasticity on hypertonia will be muscle-specific. In a study carried out with ISA on several lower limb muscles in children with CP, we identified earlier stretch reflex thresholds and less velocity-dependent activation in the hamstrings and adductor muscles when compared to the gastrocnemius [57]. Similarly, in the upper limbs of persons after stroke, Kamper et al. found earlier reflex thresholds and greater reflex responses in the finger flexors than in the elbow flexors [16]. Activation differences between muscles may be caused by differences in central and peripheral stretch reflex modulation and/or by the different morphological makeup of muscles. For example, muscle force generation is influenced by muscle-specific properties such as moment arm, cross-sectional area, and pennation angle. Differences may also reflect the dependence of a reflex response on joint position prior to stretch [58]. The rectus femoris and gastrocnemius have been found to be less sensitive when stretched from initially longer lengths [59], whilst, in the hamstrings, the opposite has been reported [17]. In biarticular muscles, the position of both joints is important when considering length dependency [58]. Therefore, subject positioning and measurement setup are important considerations when assessing spasticity and will depend on which muscle is being assessed.

#### 5.1.2. The Nonneural Component

In the classical understanding of the impairments in CP, it is generally thought that prolonged activation due to spasticity will cause fiber shortening which results in stiffer muscles [14]. Following this reasoning, spasticity can be regarded as the primary cause of hypertonia and hence the primary target for treatment. Spastic muscles have indeed been found to be smaller in volume, cross-sectional area, and muscle thickness compared to muscles of typically developing children [60]. However, contrary to the “spasticity-induced short-fiber mechanism,” studies have reported fewer, yet longer, sarcomeres in spastic muscle fibers [7]. A possible explanation may be that sarcomeres in the spastic muscle distend due to lack of growth of the muscle with bone [7]. The relationship of these changes with spasticity severity is uncertain [61].

Results from upper limb muscle biopsy studies indicate that the tensile modulus of spastic muscle cells is more than double that of muscle cells of control subjects [7]. In spastic hamstrings, increased passive stiffness is caused by increased amounts of collagen in the extracellular matrix of muscle fiber bundles [62]. Recent findings report that these changes already occur in children with CP younger than 3 years of age [54, 63] and that growth velocity, rather than spasticity, plays a crucial role in these alterations [64]. In line with these findings, muscle stiffness has been reported to increase [65], whereas spasticity decreases with age [66]. Some studies using motor-driven devices have reported higher nonneural contributions than neural ones to the torque measured during passive ankle joint rotations in children with CP [54]. Literature thus suggests that, the nonneural component is as important as, or possibly more important than spasticity.

Similar conclusions on the importance of the nonneural component in the gastroc-soleus have been derived from our own study in which the previously described work parameter significantly differed between spastic and healthy muscles but was unaffected by BTX combined with about two weeks of casting [50]. While the indirect effect of BTX injections on muscle stiffness and viscosity is not fully understood, these insights may imply that, on a group level, the nonneural component of hypertonia may not have been adequately managed.

Imaging the behavior of muscles and tendons in vivo using ultrasound (US) can provide a means to quantify muscle morphology and assess passive muscle properties [67, 68]. Dynamic US involves the real-time capture and reconstruction of images during movement. By measuring joint torque and US during muscle-tendon lengthening, the amount of torque produced per given amount of tissue length can be used to create stress-strain curves [69]. The properties of these curves can provide an indication of stiffness and viscosity in muscles and tendons. Such applications would complement instrumented spasticity assessment providing the necessary information on the nonneural components of hypertonia.

### 5.2. Active Muscle Assessments

The ultimate goal of hypertonia management in CP is to improve function, such as gait. Specific gait deviations such as early heel rise, knee flexion at initial contact, and stiff-knee gait have been attributed to lower-limb spasticity [13]. It has also been suggested that muscle stiffness may arise as a compensation for the lack of neural control [20, 70]. However, it is important to realize that gait, especially pathological gait, is multifaceted. Other impairments in CP, such as muscle weakness, involuntary coactivation, lack of muscle control, and balance problems, as well as compensatory mechanisms, will interplay with hypertonia and have an effect on gait deviations [20]. Consequently, these additional factors make ascertaining the isolated impact of hypertonia on movement abnormalities very challenging. For example, coactivation occurs when there is simultaneous motor drive to both agonist and antagonist. Involuntary coactivation is necessary for normal movement, although when excessive and inappropriate, it can also hinder it. However, coactivation is different from activation of the stretch reflex in a lengthened antagonist due to agonist contraction [11]. During activity, when both coactivation and stretch reflex activation will contribute to hypertonia, the distinction between different causes is difficult to make.

Optimal reflex activation is important in adjusting joint impedance as required for movement [20]. During activity, lack of modulation of H- and stretch reflexes may be indicative of spasticity. For example, when activated, muscles with spasticity have shown less H-reflex modulation than muscles of healthy controls [71]. Similarly, short-latency reflexes of the soleus muscles are exaggerated in adults [1] and children [70] with spasticity, while long-latency reflexes are decreased. Although reflex-related impairments can be expected to impede function in muscles with spasticity, their exact contribution to muscle hypertonia is difficult to assess.

Instead, several research groups have explored the relationship between muscle lengthening velocity and EMG during gait. Crenna (1998) was the first to describe a pathological relationship between muscle lengthening velocity and EMG during gait in children with CP. He termed this pathological relationship “dynamic spasticity” [72]. In the hamstrings, he observed that dynamic spasticity was manifested by a low muscle activation threshold at mid- and terminal swing. In the soleus, it was manifested by both a lowered threshold and increased activation during initial stance. In subsequent studies, Lamontagne et al. (2001) confirmed that increased activation in the gastrocnemius during stance and swing in adults after stroke was coincident with peak muscle lengthening velocity [73]. van der Krogt et al. (2010) found similar results in children with CP, but for the gastrocnemius during terminal swing [74]. However, in the latter study, contrary to their expectations, dynamic spasticity at high walking velocity was masked by the activation required to walk faster [74]. Similar results were found in a study carried out by our own research group, where comparable strategies to increase walking speed were employed by children with CP and typically developing children [75]. In line with this, we have recently found that reduced muscle lengthening velocity in the hamstrings during swing was associated with higher muscle activity during swing at a self-selected walking speed, but we found that these associations were no longer present at a faster walking condition [76]. Passive stiffness of the gastroc-soleus and weakness of the tibialis anterior are probably better predictors of limited dorsiflexion in terminal swing than spasticity [70]. Limited hamstrings lengthening in swing may also be explained by stiffness [77], lack of balance, and the activation necessary to decelerate knee extension [78].

To continue to analyze and understand the impact of hypertonia on gait, more information is needed on how muscles and tendons lengthen in passive conditions and during gait. Longer-than-normal Achilles tendons have been reported in children with spastic CP [79]. van der Krogt et al. (2009) found similar muscle-tendon lengthening patterns during gait in contractured and noncontractured muscles [80]. These findings may imply that contractured muscles, or muscles with altered mechanical properties, lengthen by means of a highly extensible tendon and that muscle stretch reflexes play only a minor role during walking. Ideally, to correctly investigate this, dynamic US that tracks muscle and tendon lengthening during gait should be combined with measurement of EMG.

## 6. Future Clinical Implications

In a heterogeneous condition such as CP, a personalized approach to treatment is warranted. Objective instrumented assessments that provide the treating clinician with an individualized muscle-specific hypertonia profile can be beneficial in fine-tuning a child's treatment. If research confirms that BTX primarily targets neural components whereas casting primarily influences the nonneural muscle properties, the next step would be to assess the effect of fine-tuning the combination of these two treatment modalities to a muscles' hypertonia profile. For example, if a muscle possesses a larger nonneural component, a smaller dosage of BTX and a longer casting period may be indicated. If lower BTX dosages achieve the same effect when combined with the correct period of casting, this may also be economically more advantageous.

Another important clinical application is prediction of treatment outcome. The observed variability of impairments and their response to treatment can be investigated in terms of diagnosis (unilateral versus bilateral CP), age, sex, functional level, etiology (malformations, periventricular leukomalacia, and corticosubcortical lesions), and timing of a brain lesion (pre- or postnatal). Initial findings seem to suggest that spasticity parameters collected in the hamstrings at baseline can be used to predict treatment outcome with BTX [34]. Furthermore, different lower limb muscles in children with CP have shown to have different EMG patterns during passive stretch. In a study of 54 children with CP, passive gastrocnemius and rectus femoris stretches were shown to evoke highly velocity-dependent phasic muscle activity. On the other hand, longer duration tonic activation which began at lower stretch velocities was found in the hamstrings and adductors [49]. More research is required to understand the etiology behind these differences and test the hypothesis that spasticity treatment should be muscle- and possibly also pattern-specific.

In addition to BTX, other tone reduction treatments such as intrathecal baclofen and selective dorsal rhizotomy can be fine-tuned to achieve optimal results. Studying and comparing the outcomes of the different spasticity treatments on each component of hypertonia can improve our understanding of the working mechanisms of the treatment modalities in normalizing the motor response. Longitudinal studies (in children and adults) may help to deduce the time course development of the different components of hypertonia. In turn, this will help define optimal therapeutic intervention with increasing age.

Importantly, more investigations are required to improve our understanding of the pathophysiology of hypertonia, including other primary impairments that contribute to it. Most studies attempting to quantify spasticity exclude subjects with dystonia. Unlike spasticity, dystonia is not dependent on afferent input and is not velocity dependent [11]. Therefore, the presence of dystonia can be distinguished from spasticity as it is already detectable in certain postures. Nevertheless, dystonia is affected by increasing muscle length [81]. Consequently, when spasticity and dystonia coexist, quantifying their separate contributions to hypertonia is a challenge.

Finally, a comprehensive platform for assessment should eventually consider all aspects, from muscle behavior during well-controlled passive stretches to muscle behavior during active motion. Therefore, clinical findings on the components of hypertonia assessed in an analytical manner should always be compared to measurements of activity and function. Such insight will undoubtedly help direct treatment towards its optimum goal which is to maximize a child's functional potential.

## 7. Conclusions

From the above evidence it can be concluded that hypertonia should be regarded as multifactorial and that equating all resistance to passive motion with spasticity is erroneous. Spasticity is only a contributor to this resistance, and the extent of the contribution probably differs among muscles and individuals. In this regard, the preeminent goal should be to adequately analyze the components of hypertonia. Recent developments in instrumented spasticity assessment provide a strong methodological basis on which to develop more rigorous differentiation of hypertonia into its constituents. More clinically based research is required to validate and improve our treatment modalities and to understand the effect of hypertonia on movement.
